# Short-term reproducibility of intraocular pressure and ocular perfusion pressure measurements in Chinese volunteers and glaucoma patients

**DOI:** 10.1186/s12886-016-0323-0

**Published:** 2016-08-18

**Authors:** Yanlin Gao, Bing Wan, Peiyu Li, Yan Zhang, Xin Tang

**Affiliations:** 1 Tianjin Eye Hospital, Tianjin Key Laboratory of Ophthalmology and Vision Science, Clinical College of Ophthalmology, Tianjin Medical University, 4, Gansu Road, Heping District, Tianjin, 300020 People’s Republic of China; 2 Department of Laboratory, Central Hospital Affiliated to Shenyang Medical College, Shenyang, China; 3 Department of Ophthalmology, the Second Hospital Affiliated to Dalian Medical University, Dalian, China

## Abstract

**Background:**

To evaluate the short-term reproducibility of diurnal intraocular pressure (IOP) and ocular perfusion pressure (OPP) measurements in normal volunteers, untreated normal-tension glaucoma (NTG) and primary open-angle glaucoma (POAG) patients.

**Methods:**

Fifty-four healthy volunteers (control group), 67 NTG patients and 54 POAG patients were recruited. The IOPs of both eyes were measured with a Goldmann applanation tonometer at 3-h intervals over 2 consecutive days. Blood pressure (BP) measurements were collected at the same times. The mean IOP/OPP, peak IOP/OPP, trough IOP/OPP and IOP/OPP fluctuations on each day were also calculated. The intraclass correlation coefficients (ICCs) were used to evaluate the reproducibilities.

**Results:**

In the control group, the ICCs of mean IOP, peak IOP, trough IOP and IOP fluctuation were 0.921, 0.889, 0.888, and 0.661, respectively, and the ICCs of the mean OPP, peak OPP, trough OPP and OPP fluctuations were 0.962, 0.918, 0.953, and 0.680, respectively. In the NTG group, the ICCs of the mean IOP, peak IOP, trough IOP and IOP fluctuation were 0.862, 0.741, 0.798, and 0.290, respectively, and the ICCs of the mean OPP, peak OPP, trough OPP and OPP fluctuations were 0.947, 0.828, 0.927, and −0.008, respectively. In the POAG group, the ICCs of the mean IOP, peak IOP, trough IOP and IOP fluctuation were 0.857, 0.666, 0.808, and 0.546, respectively, and the ICCs of the mean OPP, peak OPP, trough OPP and OPP fluctuation were 0.934, 0.842, 0.910, and 0.093, respectively.

**Conclusion:**

The IOP measurements within a single day were not highly reproducible in the short-term. The normal volunteers exhibited better IOP and OPP reproducibilities than the glaucoma patients. The IOP and OPP fluctuations could not be accurately evaluated based on the IOP or OPP measurements within a single day.

## Background

Elevated intraocular pressure (IOP) is an identified risk factor for the progression of glaucoma [1, 2]. Moreover, IOP fluctuations [3] and the long-term mean IOP [4] are significantly correlated with the development of glaucoma. Glaucoma treatment focuses on IOP reduction. It is very important to assess the IOP level before initiating anti-glaucoma treatment.

Patients with normal-tension glaucoma (NTG) exhibit significantly greater reductions in nocturnal blood pressure (BP) than healthy people [5, 6], and vascular factors might be associated with the development of glaucoma [6–9]. IOP [10] and BP [10, 11] change over time and exhibit inherent circadian rhythms. The ocular perfusion pressure (OPP) is calculated from the IOP and BP. Lower diastolic OPP has been associated with glaucoma in previous population-based studies [12, 13]. The mean circadian OPP fluctuation is a consistent clinical risk factor for the severity and development of NTG [6–8]. Relative changes in the diurnal mean OPP have also been identified as a risk factor for the diagnosis of primary open-angle glaucoma (POAG) [9].

Twenty-four-hour IOP measurements are widely used in clinical and research practices. We typically measure IOPs at various time points within a single day based on convenience, time and financial cost. IOP values at the same time points are not always stable. The reproducibilities of 24-h IOP patterns are controversial [14–19]. If 24-h IOP patterns are not highly reproducible, IOP measurements over 2 consecutive days or over the long term are necessary to assess the IOP condition.

In this study, normal volunteers, untreated NTG patients and POAG patients underwent IOP and OPP measurements over 2 consecutive days. We analysed the short-term reproducibilities of the IOP and OPP measurements in the 3 groups.

## Methods

### Subjects

The study adhered to the tenets of the Declaration of Helsinki and was approved by the institutional review board and Ethics Committee of Tianjin Eye Hospital. Informed consent was obtained from each subject. The subjects were recruited in the Tianjin Eye Hospital, Tianjin, China. All subjects were free of treatments with anti-glaucoma medications for at least 4 weeks before the IOP measurements. Subjects with systemic hypertension were excluded. And subjects with systemic administration affected BP or IOP were excluded. None of the subjects had any history of ocular surgery or trauma.

None of the normal volunteers had family histories of glaucoma. The IOP values of the normal volunteers ranged from 8 mmHg to 21 mmHg. The volunteers underwent complete ophthalmic examinations and exhibited no signs of ophthalmic disease.

The NTG patients were characterized by IOPs ≤ 21 mmHg at all time points, glaucomatous visual field (VF) defects, optic disc damage, an open angle of normal appearance, and the absence of secondary causes for glaucomatous optic disc damage.

The POAG patients were characterized by IOPs > 21 mmHg at all time points, glaucomatous VF defects, optic disc damage, an open angle of normal appearance, and the absence of secondary causes of glaucomatous optic disc damage.

All subjects underwent a complete ophthalmic examination including central corneal thickness (CCT) measurements (Pentacam, Oculus, Inc., Wetzlar, Germany) and VF tests using a Humphrey Field Analyzer 750i (30–2 program; Carl Zeiss Meditec, Inc., Dublin, California).

### IOP measurements

The IOPs of both eyes was measured over 2 consecutive days. All subjects were in a sitting position, and the IOPs were measured with a Goldmann applanation tonometer (GAT, Carl Zeiss, Inc., Jena, Germany) at 3-h intervals from 0600 to 2400 h. Two experienced doctors were in charge of the IOP measurements. One doctor measured the IOPs on day 1 (6:00–24:00), and the other doctor, masked to the IOP data on day 1, measured the IOPs on day 2 (6:00–24:00). The nocturnal period was from 21:00 to 06:00 [20]. In the control group, the IOP data from the eye with the better mean deviation (MD) value of visual field was selected. In the glaucoma patients, the IOP data from the eye with the greater visual field defect based on the MD values was selected.

The mean IOP was defined as the average IOP across all measurements over 2 consecutive days (14 time points). The IOPs at each time point were defined as the average IOPs at same time point across all subjects within each group. The mean IOPs on days 1 and day 2 were defined as the average IOPs of all measurements during each day (6:00–24:00, 7 time points). The peak IOP, trough IOP and IOP fluctuation (the peak IOP minus the trough IOP) were also calculated from the IOP measurements during each day. For each subject, the IOPs at each time point were calculated as the average IOPs over the 2 consecutive days. Furthermore, the time points of the peak IOPs, the maximum daytime IOPs and maximum nighttime IOPs were recorded.

### OPP calculation

The systolic BP (SBP), diastolic BP (DBP) and heart rate (HR) were also measured at same time points from 06:00 to 24:00 h. All subjects were in a sitting position and kept calm for at least 5 min. The BP and HR were measured on the upper left arm with an automated sphygmomanometer (OMRON HBP-1300, OMRON Healthcare (China) Co., Ltd.). The mean SBP, DBP, and HR were defined as the averages of the data over 2 consecutive days (14 time points). The OPPs at each time point were calculated as follows: OPP = 2/3× [DBP + {1/3 × (SBP − DBP)}] − IOP [21]. The main parameters of the OPP were calculated with the same methods used for the IOP.

### Statistical analysis

The gender, eye (right/left), and MD values of the 3 groups were analysed using Kruskal-Wallis H test. The age, CCT, IOP at diagnosis, mean IOP, mean OPP, mean SBP, mean DBP and mean HR were analysed using one-way analyses of variance (ANOVA). The reproducibilities over the 2 consecutive days were elevated with the intraclass correlation coefficients (ICCs) and Bland-Altman Plots. The ICC indicates the proportion of variance in a measurement that is due to differences among subjects. The α level (type I error) was set at 0.05. An ICC ≥ 0.75 was considered indicative of excellent reproducibility, an ICC of 0.4 ≤ ICC < 0.75 was considered indicative of fair reproducibility, and an ICC of ICC < 0.4 was considered indicative of poor reproducibility [22]. Negative ICC values indicate greater within-subject variability than between-subject variability and represent an agreement that is below that expected by chance alone [17]. The statistical analyses were performed with SPSS (version 16.0; SPSS Inc., Chicago, IL). Bland-Altman Plots were created with Medcalc (Version 11.4.2.0; Medcalc Software Inc., Mariakerke, Belgium). *P* < 0.05 was considered statistically significant.

## Results

### Subject characteristics

Fifty-four normal volunteers (control group), 67 NTG patients and 54 POAG patients were recruited, and the characteristics of each group are shown in Table 1. The POAG group exhibited a significantly higher mean IOP over the 2 consecutive days (28.3 ± 2.1 mmHg) than the control (14.3 ± 1.9 mmHg) and NTG groups (13.9 ± 1.6 mmHg, *P* < 0.001). The mean IOPs over 2 consecutive days exhibited no significant difference between the control and NTG groups (*P* = 0.271). The POAG group exhibited a significantly lower mean OPP over the 2 consecutive days (29.6 ± 4.5 mmHg) than the control (44.7 ± 4.8 mmHg) and NTG groups (43.5 ± 5.2 mmHg, *P* < 0.001). The mean OPP over 2 consecutive days exhibited no significant difference between the control and NTG groups (*P* = 0.186). The ages, eyes (right/left), genders, CCTs, mean SBPs, mean DBPs and mean HRs over the 2 consecutive days exhibited no significant differences within the 3 groups.

**Table 1:** Characteristics of Subjects

Characteristic	Control	NTG	POAG	*P* value
Number	54	67	54	
Gender (Male/Female)^a^	31/23	37/30	32/22	0.965
Eye (Right/left)^a^	32/22	34/33	26/28	0.479
Age(yrs)	50.7 ± 14.0	52.2 ± 13.6	51.4 ± 11.7	0.830
IOP at diagnosis (mmHg)	15.5 ± 2.5	15.2 ± 2.1	28.6 ± 3.1	<0.001
Mean deviation (dB)^a^	−0.51 ± 1.0	−9.32 ± 4.91	−9.60 ± 5.52	<0.001
CCT (μm)	549.2 ± 30.5	542.6 ± 31.3	555.0 ± 26.7	0.064
Mean IOP (mmHg)	14.3 ± 1.9	13.9 ± 1.6	28.3 ± 2.1	<0.001
Mean OPP (mmHg)	44.7 ± 4.8	43.5 ± 5.2	29.6 ± 4.5	<0.001
Mean SBP (mmHg)	118.7 ± 9.4	116.3 ± 10.2	117.4 ± 9.2	0.395
Mean DBP (mmHg)	73.4 ± 6.8	70.9 ± 7.6	71.7 ± 6.0	0.139
Mean HR	68.8 ± 8.1	68.6 ± 7.3	68.4 ± 7.1	0.969

*NTG* normal tension glaucoma, *POAG* primary open-angle glaucoma, *IOP* intraocular pressure; *CCT* central corneal thickness, *OPP* ocular perfusion pressure; *SBP* systolic blood pressure, *DBP* diastolic blood pressure, *HR* heart rate

Data are expressed as mean ± standard deviation

^a^Kruskal-Wallis test; other parameters were analyzed using one-way analysis of variance

### ICC estimates of the IOPs at each time point

The ICC estimates of the IOPs at each time point across the 2 days are presented in Table 2. In the control group, the ICCs ranged from 0.688 (15:00) to 0.876 (6:00). The ICC values exhibited fair to excellent reproducibility. In the NTG group, the ICCs ranged from 0.347 (15:00) to 0.762 (21:00) and indicated large variations in reproducibility from poor to excellent at different time points. In the POAG group, the ICCs ranged from 0.595 (21:00) to 0.787 (9:00 and 12:00), and these ICC values indicated fair to excellent reproducibility.

**Table 2:** Intraclass Correlation Coefficient Estimates of Intraocular Pressure at Each Time Point

Time point		Control			NTG			POAG	
	Day 1	Day 2	ICC^a^	Day 1	Day 2	ICC^a^	Day 1	Day 2	ICC^a^
6:00	15.5 ± 2.6	14.8 ± 2.7	0.876	14.6 ± 2.3	13.9 ± 2.3	0.696	29.1 ± 3.1	28.6 ± 2.9	0.744
9:00	14.2 ± 2.5	14.6 ± 2.5	0.815	13.7 ± 2.0	13.8 ± 2.4	0.646	28.5 ± 3.1	28.1 ± 3.0	0.787
12:00	14.9 ± 2.6	15.1 ± 2.6	0.802	14.4 ± 2.1	14.6 ± 2.1	0.694	29.2 ± 3.0	29.1 ± 3.0	0.787
15:00	14.0 ± 2.1	14.0 ± 2.5	0.688	14.0 ± 2.0	13.8 ± 2.1	0.347	28.3 ± 3.1	27.9 ± 2.6	0.752
18:00	14.7 ± 2.2	14.8 ± 2.2	0.736	14.6 ± 2.2	14.4 ± 2.0	0.710	29.2 ± 2.9	28.7 ± 2.4	0.708
21:00	13.1 ± 2.4	13.2 ± 2.3	0.756	13.5 ± 2.5	13.2 ± 2.2	0.762	27.6 ± 3.3	27.3 ± 2.4	0.595
24:00	13.3 ± 2.6	13.3 ± 2.2	0.714	13.0 ± 2.3	13.0 ± 2.4	0.523	27.6 ± 2.7	27.2 ± 3.2	0.656

Data are expressed as mean ± standard deviation

*NTG* normal tension glaucoma, *POAG* primary open-angle glaucoma, *ICC* intraclass correlation coefficient

^a^All ICC measurements, *P* < 0.001

### ICC estimates of the OPPs at each time point

The ICC estimates of the OPPs at each time point across the 2 days are presented in Table 3. In the control group, the ICCs ranged from 0.784 (24:00) to 0.896 (6:00) and indicated excellent reproducibility. In the NTG group, the ICCs ranged from 0.720 (18:00) to 0.891 (21:00). Most of these ICC values indicated excellent reproducibility, and only one value (0.720, 18:00) indicated fair reproducibility. In the POAG group, the ICCs ranged from 0.714 (18:00) to 0.878 (9:00), and most of these ICC values indicated excellent reproducibility.

**Table 3:** Intraclass Correlation Coefficient Estimates of Ocular Perfusion Pressure at Each Time Point

Time point		Control			NTG			POAG	
	Day 1	Day 2	ICC^a^	Day 1	Day 2	ICC^a^	Day 1	Day 2	ICC^a^
6:00	45.6 ± 6.5	42.8 ± 6.7	0.896	42.9 ± 6.8	42.3 ± 6.1	0.841	30.7 ± 6.0	28.7 ± 5.1	0.857
9:00	44.9 ± 6.0	43.5 ± 5.4	0.787	44.0 ± 5.8	42.7 ± 6.2	0.880	29.6 ± 5.0	28.1 ± 5.3	0.878
12:00	43.4 ± 6.7	43.4 ± 6.8	0.889	41.9 ± 5.4	43.0 ± 5.6	0.797	28.6 ± 5.8	28.4 ± 5.5	0.734
15:00	44.6 ± 6.2	43.7 ± 6.1	0.791	42.7 ± 6.8	42.6 ± 6.4	0.832	29.1 ± 5.7	29.3 ± 5.8	0.805
18:00	46.1 ± 6.6	45.5 ± 6.3	0.882	45.2 ± 6.5	43.9 ± 6.7	0.720	30.3 ± 6.2	29.2 ± 6.1	0.714
21:00	45.8 ± 5.8	46.0 ± 5.7	0.867	43.7 ± 7.0	44.3 ± 5.2	0.891	30.9 ± 6.0	30.1 ± 5.4	0.818
24:00	45.5 ± 5.7	45.9 ± 5.5	0.784	44.5 ± 6.5	45.1 ± 5.6	0.832	31.3 ± 5.3	30.3 ± 5.3	0.757

Data are expressed as mean ± standard deviation

*NTG* normal tension glaucoma, *POAG* primary open-angle glaucoma, *ICC* intraclass correlation coefficient

^a^All ICC measurements, *P* < 0.001

### ICC estimates for the main parameters of diurnal curves

**Fig. 1:**
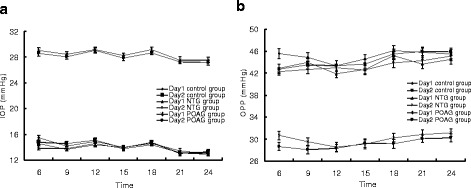
Diurnal Curves of the 3 groups. The diurnal IOP (**a**) and diurnal OPP curves (**b**) were plotted according to the IOP and OPP data at each time point. IOPs and OPPs changed with time and showed a typical circadian rhythm. The peak IOPs did not occur at same time points over the 2 days in the normal volunteers or glaucoma patients. The peak OPPs occurred at same time points (24:00) only in the POAG patients

The diurnal IOP and diurnal OPP curves were plotted according to the IOP and OPP data at each time point (Fig. 1). The mean IOP/OPP, peak IOP/OPP, trough IOP/OPP and IOP/OPP fluctuation values were calculated from the diurnal curves. The ICC estimates of these parameters for the 2 days are presented in Table 4.

**Table 4:** Intraclass Correlation Coefficient Estimates for the Main Parameters in diurnal curves

Parameters		Control			NTG			POAG	
	Day 1	Day 2	ICC^a^	Day 1	Day 2	ICC^a^	Day 1	Day 2	ICC^a^
Mean IOP	14.3 ± 2.0	14.3 ± 2.0	0.921	14.0 ± 1.7	13.8 ± 1.7	0.862	28.5 ± 2.4	28.1 ± 2.0	0.857
Peak IOP	16.8 ± 2.2	16.4 ± 2.2	0.889	16.3 ± 1.9	16.0 ± 1.7	0.741	31.2 ± 2.4	31.1 ± 2.1	0.666
Trough IOP	12.0 ± 2.0	12.0 ± 2.0	0.888	11.8 ± 1.7	11.7 ± 2.1	0.798	25.8 ± 2.6	25.3 ± 2.3	0.808
IOP Fluctuation	4.8 ± 1.4	4.3 ± 1.4	0.661	4.5 ± 1.4	4.4 ± 1.5	0.290	5.4 ± 1.8	5.7 ± 2.0	0.546
Mean OPP	45.1 ± 5.3	44.4 ± 5.2	0.962	43.6 ± 5.5	43.4 ± 5.1	0.947	30.1 ± 4.7	29.2 ± 4.5	0.934
Peak OPP	49.8 ± 5.5	49.3 ± 5.7	0.918	48.7 ± 6.2	48.2 ± 5.4	0.828	34.8 ± 5.3	34.1 ± 4.6	0.842
Trough OPP	40.1 ± 5.9	39.3 ± 5.6	0.953	38.7 ± 5.5	38.5 ± 5.4	0.927	25.2 ± 4.8	24.7 ± 4.7	0.910
OPP Fluctuation	9.7 ± 3.4	10.0 ± 3.0	0.680	10.0 ± 4.4	9.7 ± 3.4	−0.008	9.7 ± 3.3	9.5 ± 2.8	0.093

Data are expressed as mean ± standard deviation

*IOP* intraocular pressure, *OPP* ocular perfusion pressure, *ICC* intraclass correlation coefficient

^a^All ICC measurements, *P* < 0.001

In the control group, the IOP reproducibility was greatest for the mean IOP (ICC = 0.921) followed by the peak IOP (ICC = 0.889) and the trough IOP (ICC = 0.888). The IOP fluctuation exhibited the lowest reproducibility (ICC = 0.661). The reproducibility of the OPP was highest for the mean OPP (ICC = 0.962) followed by the trough OPP (ICC = 0.953) and the peak OPP (ICC = 0.918). The OPP fluctuation exhibited the lowest reproducibility (ICC = 0.680).

In the NTG group, the IOP reproducibility was highest for the mean IOP (ICC = 0.862) followed by the trough IOP (ICC = 0.798) and the peak IOP (ICC = 0.741). The IOP fluctuation exhibited the lowest reproducibility (ICC = 0.290). The reproducibility of the OPP was highest for the mean OPP (ICC = 0.947) followed by the trough OPP (ICC = 0.927) and the peak OPP (ICC = 0.828). The OPP fluctuation exhibited the lowest reproducibility (ICC = −0.008).

In the POAG group, the IOP reproducibility was highest for the mean IOP (ICC = 0.857) followed by the trough IOP (ICC = 0.808) and the peak IOP (ICC = 0.666). The IOP fluctuation exhibited the lowest reproducibility (ICC = 0.546). The reproducibility of the OPP was highest for the mean OPP (ICC = 0.934) followed by the trough OPP (ICC = 0.910) and the peak OPP (ICC = 0.842). The OPP fluctuation exhibited the lowest reproducibility (ICC = 0.093).

For each group, the mean IOPs and mean OPPs exhibited the greatest reproducibilities, whereas the IOP and OPP fluctuations elicited the poorest reproducibilities.

### Bland-Altman plots of main parameters of the diurnal curves

**Fig. 2:**
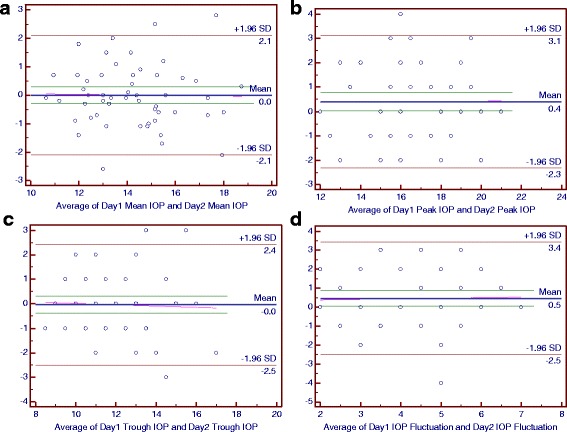
The Bland-Altman Plots for Intraocular Pressure (IOP) in the Control Group. Part **a**, **b**, **c** and **d** respectively reflected the individual test-retest difference conditions of mean IOP, peak IOP, trough IOP and IOP fluctuation

**Fig. 3:**
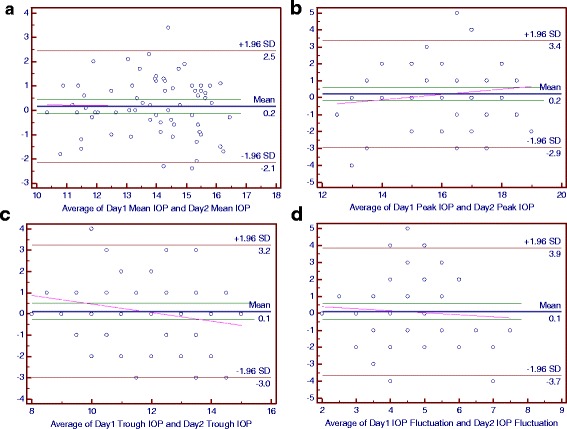
The Bland-Altman Plots for Intraocular Pressure (IOP) in the Normal-Tension Glaucoma Group. Part **a**, **b**, **c** and **d** respectively reflected the individual test-retest difference conditions of mean IOP, peak IOP, trough IOP and IOP fluctuation

**Fig. 4:**
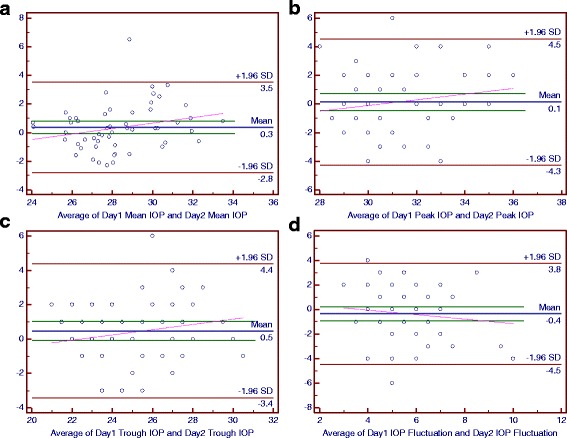
The Bland-Altman Plots for Intraocular Pressure (IOP) in the Primary Open-Angle Glaucoma Group. Part **a**, **b**, **c** and **d** respectively reflected the individual test-retest difference conditions of mean IOP, peak IOP, trough IOP and IOP fluctuation

**Fig. 5:**
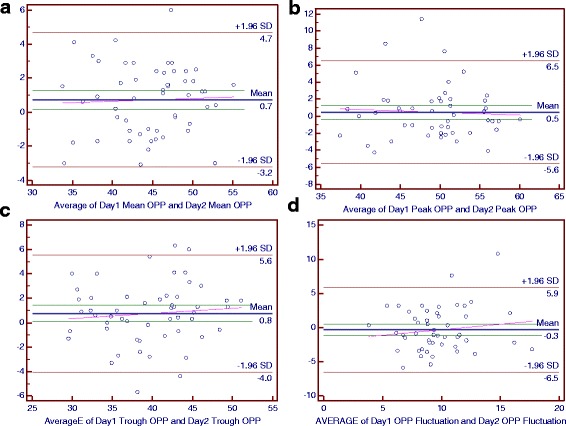
The Bland-Altman Plots for Ocular Perfusion Pressure (OPP) in the Control Group. Part **a**, **b**, **c** and **d** respectively reflected the individual test-retest difference conditions of mean OPP, peak OPP, trough OPP and OPP fluctuation

**Fig. 6:**
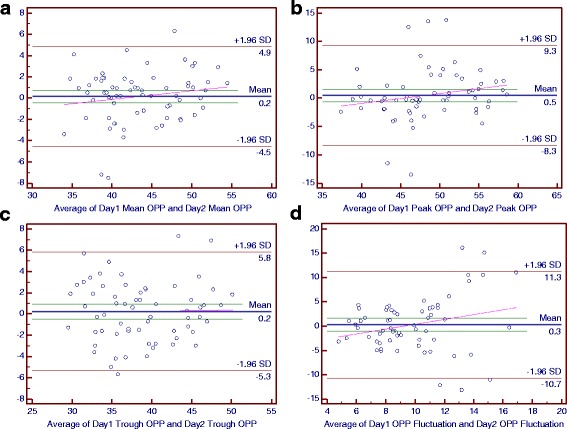
The Bland-Altman Plots for Ocular Perfusion Pressure (OPP) in the Normal-Tension Glaucoma Group. Part **a**, **b**, **c** and **d** respectively reflected the individual test-retest difference conditions of mean OPP, peak OPP, trough OPP and OPP fluctuation

**Fig. 7:**
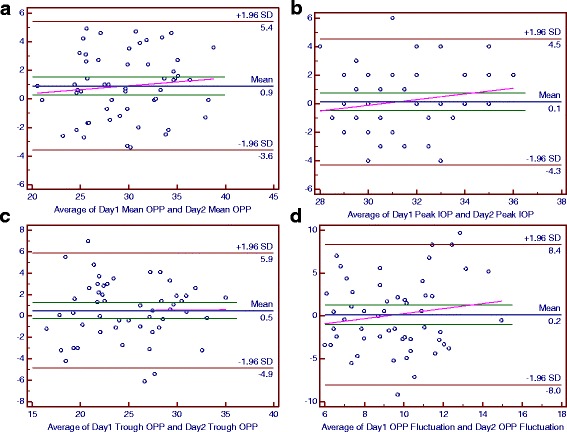
The Bland-Altman Plots for Ocular Perfusion Pressure (OPP) in the Primary Open-Angle Glaucoma Group. Part **a**, **b**, **c** and **d** respectively reflected the individual test-retest difference conditions of mean OPP, peak OPP, trough OPP and OPP fluctuation

Figure 2 to 7 present Bland-Altman plots comparing the main parameters of the individuals over 2 days in the control, NTG and POAG groups.

For the control group, the mean differences between 2 days were 0 mmHg for the mean IOP and trough IOP, 0.4 mmHg for the peak IOP, 0.5 mmHg for the IOP fluctuation and peak OPP, 0.7 mmHg for the mean OPP, 0.8 mmHg for the trough OPP and −0.3 mmHg for the OPP fluctuation (Figs. 2 and 3).

For the NTG group, the mean differences between 2 days were 0.1 mmHg for the trough IOP and IOP fluctuation, 0.2 mmHg for the mean IOP, peak IOP, mean OPP and trough OPP, 0.3 mmHg for the OPP fluctuation and 0.5 mmHg for the peak OPP (Figs. 4 and 5).

For the POAG group, the mean differences between the 2 days were 0.1 mmHg for the peak IOP and peak OPP, 0.2 mmHg for the OPP fluctuation, 0.3 mmHg for the mean IOP, 0.5 mmHg for the trough IOP and trough OPP, 0.9 mmHg for the mean OPP and −0.4 mmHg for the IOP fluctuation (Figs. 6 and 7).

### Test-retest differences in the main parameters of the diurnal curves

The test-retest differences in the main parameters in the different intervals were calculated and are presented in Table 5 and 6.

**Table 5:** Proportions of Parameter Test-Retest Differences within Different Intervals between Two Days

Interval		Control				NTG				POAG		
	Mean IOP	Peak IOP	Trough IOP	IOP Fluctuation	Mean IOP	Peak IOP	Trough IOP	IOP Fluctuation	Mean IOP	Peak IOP	Trough IOP	IOP Fluctuation
≤1 mmHg	70.4	74.1	79.6	64.8	62.7	70.1	68.7	58.2	59.3	44.4	57.4	55.6
≤2 mmHg	92.6	92.6	94.4	88.9	91.0	88.1	85.1	80.6	81.5	77.8	74.1	75.9
≤3 mmHg	100	98.1	98.1	98.1	98.5	95.5	98.5	92.5	94.4	85.2	94.4	88.9
≤4 mmHg		100	100	100	100	98.5	100	98.5	98.1	98.1	98.1	98.1
≤5 mmHg						100		100	98.1	98.1	98.1	98.1
≤6 mmHg									98.1	100	100	100
≤7 mmHg									100			

Data are expressed as percentage

*IOP* intraocular pressure, *NTG* normal tension glaucoma, *POAG* primary open-angle glaucoma

**Table 6:** Proportions of Parameter Test-Retest Differences within Different Intervals between Two Days

Interval		Control				NTG				POAG		
	Mean OPP	Peak OPP	Trough OPP	OPP Fluctuation	Mean OPP	Peak OPP	Trough OPP	OPP Fluctuation	Mean OPP	Peak OPP	Trough OPP	OPP Fluctuation
≤1 mmHg	24.1	31.5	31.5	16.7	38.8	31.3	25.4	14.9	31.5	24.1	24.1	13.0
≤2 mmHg	66.7	63.0	63.0	42.6	70.1	52.2	53.7	32.8	55.6	48.1	53.7	33.3
≤3 mmHg	88.9	81.5	79.6	63.0	85.1	65.7	74.6	46.3	71.4	63.0	72.2	55.6
≤4 mmHg	94.4	85.2	87.0	88.9	92.5	77.6	85.1	61.2	87.0	74.1	85.2	64.8
≤5 mmHg	98.1	88.9	92.6	92.6	95.5	82.1	94.0	71.6	100	75.9	92.6	75.9
≤6 mmHg	100	94.4	98.1	96.3	95.5	89.6	97.0	82.1		83.3	96.3	87.0
≤7 mmHg		94.4	100	96.3	97.0	91.0	98.5	85.1		92.6	100	90.7
≤8 mmHg		96.3		98.1	100	92.5	100	85.1		100		92.6

Data are expressed as percentage

*OPP* ocular perfusuion pressure, *NTG* normal tension glaucoma, *POAG* primary open-angle glaucoma

The test-retest difference in the mean IOPs of the control, NTG, and POAG groups fell within ±2 mmHg in 92.6, 91.0 and 81.5 % of the subjects, respectively. The corresponding test-retest difference in the peak IOPs of the 3 group fell within ±2 mmHg in 92.6, 88.1 and 77.8 % of the subjects. The corresponding test-retest differences in the trough IOPs fell within ±2 mmHg in 94.4, 85.1 and 74.1 % of the subjects. The test-retest differences in the IOP fluctuations in the control, NTG, and POAG groups fell within ±2 mmHg in 88.9, 80.6 and 75.9 % of the subjects, respectively.

The test-retest differences in the mean OPPs of the control, NTG, and POAG groups fell within ±5 mmHg in 98.1, 95.5 and 100 % of the subjects, respectively. The corresponding test-retest differences in the peak OPPs fell within ±5 mmHg in 88.9, 82.1 and 75.9 % of the subjects. The corresponding test-retest differences in the trough OPPs fell within ±5 mmHg in 92.6, 94.0 and 92.6 % of the subjects. The test-retest differences in the IOP fluctuations in the control, NTG, and POAG groups fell within ±5 mmHg in 92.6, 71.6 and 75.9 % of the subjects, respectively.

### Daytime vs. nighttime IOPs/OPPs

The distributions of the peak IOP/OPP times are illustrated in Table 7. The peak IOP time points with the greatest frequencies were 6:00 for the control group (46.3 %), 18:00 for the NTG group (34.3 %) and 12:00 for the POAG group (29.6 %). The times with the greatest frequencies of peak OPPs were 18:00 in the control group (20.4 %), 24:00 in the NTG group (28.4 %) and 24:00 in the POAG group (27.8 %).

**Table 7:** Peak Intraocular Pressure and Ocular Perfusion Pressure (mmHg)

	Time point	Patients with peak IOP at time point (%)^a^			Patients with peak OPP at time point (%)^a^		
		Control	NTG	POAG	Control	NTG	POAG
Daytime	0900	14.8	16.4	20.4	11.1	10.4	5.6
	1200	25.9	31.3	29.6	13.0	1.5	7.4
	1500	11.1	9.0	9.3	9.3	6.0	14.8
	1800	20.4	34.3	24.1	20.4	28.4	9.3
Nighttime	2100	3.7	9.0	1.9	18.5	13.4	14.8
	2400	3.7	9.0	9.3	13.0	28.4	27.8
	0600	46.3	31.3	25.9	14.8	11.9	20.4

Data are expressed as percentage

*IOP* intraocular pressure, *OPP* ocular perfusion pressure, *NTG* normal tension glaucoma, *POAG* primary open-angle glaucoma

^a^The same peak pressure may have occurred at more than one time point

Table 8 illustrates how well the maximum daytime pressures predicted the nighttime measurements at various pressure levels. The table indicates that the nighttime pressures of 76.0 % of the normal volunteers, 71.7 % of the NTG patients and 59.3 % of the POAG patients were within 1.0 mmHg of the maximum daytime IOP readings. Furthermore, the nighttime pressures of 77.8 % of the normal volunteers, 62.7 % of the NTG patients and 66.6 % of the POAG patients were within 2.0 mmHg of the maximum daytime OPP readings.

**Table 8:** Maximum Daytime Pressures Measured within Different Intervals of Maximum Nighttime Pressures

Nighttime elevation above daytime peak pressure (mmHg)	Per cent of daytime IOP < than indicated level in column 1 (%)			Per cent of daytime OPP < than indicated level in column 1 (%)		
	Control	NTG	POAG	Control	NTG	POAG
≤0	51.9	44.8	38.9	53.7	47.8	40.7
+1	24.1	26.9	20.4	9.3	1.5	14.8
+2	9.3	16.4	33.3	14.8	13.4	11.1
+3	11.1	7.5	3.7	13	14.9	13
+4	1.9	4.8	3.7	5.6	11.9	7.4
≥5	1.9	0	0	3.7	10.4	13.0

Data are expressed as percentage

*IOP* intraocular pressure, *OPP* ocular perfusion pressure, *NTG* normal tension glaucoma, *POAG* primary open-angle glaucoma

The POAG patients had significantly greater nighttime OPPs (30.3 ± 5.1 mmHg) than daytime OPPs (29.1 ± 5.1 mmHg, *P* = 0.020), whereas the control and NTG group exhibited no differences between the daytime and nighttime OPPs.

## Discussion

In this study, we collected IOP measurements over 2 consecutive days that revealed the following findings: (1) POAG group exhibited larger IOP fluctuations than the control and NTG groups; (2) the POAG group exhibited significantly lower OPPs than the control and NTG groups; (3) the control group exhibited better IOP reproducibility across the time points than the glaucoma groups; (4) the OPPs across the time points in each group exhibited a tendency towards excellent reproducibility; (5) the mean and trough IOPs of each group exhibited excellent reproducibilities, the peak IOP of the control group exhibited better excellent reproducibility than those of the glaucoma groups, and the IOP fluctuations exhibited fair or poor reproducibilities; (6) the mean OPP, peak OPP and trough OPP exhibited excellent reproducibilities, while the OPP fluctuations exhibited fair or poor reproducibilities; and (7) the nighttime readings of the majority of the subjects were within 1.0 mmHg of the maximum daytime IOP reading and 2.0 mmHg of the maximum daytime OPP.

Typical patterns of diurnal IOP and OPP fluctuations were observed in the present study [21]. The peak IOPs did not occur at same time points over the 2 days in the normal volunteers or glaucoma patients. The peak OPPs occurred at same time points (24:00) only in the POAG patients. The greatest frequencies of peak IOPs occurred at different time points, and the greatest highest frequencies of peak OPPs occurred at same time point (24:00) in the glaucoma patients. Quaranta et al. reported that the great majority of untreated glaucoma patients exhibit their peak IOP during the daytime, and the highest nighttime IOP values of approximately 70 % of patients are within 1.0 mmHg of the highest daytime IOP values [20]. In this study, some subjects exhibited peak IOPs in the morning (06:00), and the highest nighttime IOPs of 59.3 % of the POAG patients were within 1 mmHg of the highest daytime IOPs. The peak IOPs most frequently occurred outside of office hours. Furthermore, the characteristics of the 24-h IOP curves were not correctly predicted based on the IOP data collected during office hours [23]. The peak IOPs were not highly reproducible, and the peak IOPs times were quite different, and these differences may be attributable to differences in the body positions, tonometers and circadian CCT fluctuations.

The short- and long-term reproducibilities of IOP measurements have been evaluated in some studies. Realini et al. measured the IOPs of healthy individuals with a GAT at 2 h intervals from 8:00 to 20:00 during 2 visits spaced 1 week apart. The eyes of the healthy individuals did not exhibit a sustained and reproducible diurnal IOP pattern across the 2 visits [14]. Later, Realini et al. measured the IOPs of treated POAG patients at the same time points. The treated POAG patients did not manifest a repeatable diurnal IOP pattern across 2 visits spaced 1 week apart [15]. Hatanaka et al. measured the IOPs of ocular hypertension (OHT) and POAG patients with a GAT at 8:00, 11:00, 14:00 and 16:00 over 2 consecutive days. The diurnal mean IOP, peak IOP and trough IOP exhibited excellent reproducibility, but the IOP fluctuation exhibited fair reproducibility [16]. Song et al. measured the IOPs of healthy volunteers with a GAT and a Tono-Pen AVIA tonometer every 3 h once per week for 5 consecutive weeks. The maximum IOP and minimum IOP as measured with the GAT exhibited excellent reproducibility, but the IOP fluctuation exhibited poor reproducibility [17]. Aptel et al. measured the IOPs of POAG patients with a GAT at 9:00, 10:00, 11:00, 12:00, 14:00, 15:00, 16:00 and 17:00 over 4 visits in 6 months. The POAG patients did not exhibit a reproducible diurnal IOP pattern from month to month [18]. Xu et al. measured the IOPs of POAG and OHT patients with a non-contact tonometer at 2-h intervals over 2 consecutive days. The IOPs at the different time points generally exhibited fair or poor reproducibility, and the 24-h IOP curve of a single day was not highly reproducible in the short-term. The POAG group exhibited excellent reproducibility in terms of the mean IOP but fair reproducibility in terms of the peak IOP and poor reproducibility in terms of the IOP fluctuation. The OHT group exhibited excellent reproducibilities of the mean IOP and peak IOP but fair reproducibility of the IOP fluctuation [19]. Our data are partly consistent with those of previous studies, and the differences might be attributable to different sample sizes, study populations, IOP measurement time points, body positions, measurement intervals and types of tonometer. Furthermore, the CCT fluctuates with time and exhibits a circadian fluctuation [24]. However, the 24-h IOP measurements were not influenced by the CCT fluctuations in either the both treated or untreated glaucoma patients [25]. We did not measure the CCTs at each time point; thus, we were unable to evaluate whether the CCT fluctuations influenced the reproducibilities of the 24-h IOPs or OPPs.

The mean IOP change between the short- and long-term periods and the short-term peak IOP were associated with progression of glaucoma [26]. Small mean changes (i.e., 1 mmHg for the mean, 2 mmHg for the peak, and 0.5 mmHg for the fluctuations) elicited major changes in the single measurements [26]. In this study, in approximately 60 % the glaucoma patients, the test-retest differences in the IOPs fell within ±1 mmHg. Further studies are needed to assess the influence of IOP reproducibility on the progression of glaucoma.

Song et al. also calculated the diurnal OPPs of young volunteers every 3 h once per week for 5 consecutive weeks. The maximum OPP, minimum OPP and OPP fluctuation values exhibited excellent, fair and poor reproducibilities, respectively [17]. In this study, the peak OPP, trough OPP and OPP values at the time points exhibited good reproducibilities in the normal volunteers, but the OPP fluctuations exhibited poor reproducibility. Our data are partially consistent with the findings of Song et al. The differences might be due to the different sample sizes (54 subjects vs. 10 subjects), study populations (Chinese vs. young female Koreans) and measurement intervals (2 consecutive days vs. 5 consecutive weeks).

In this study, the POAG group exhibited a significantly lower OPP than the NTG patients and normal volunteers. A reduction of OPP is indicative of a reduction in the vascular flow to the optic nerve and could lead to glaucomatous optic nerve damage [13, 27]. Sehi et al. reported that a relative change in the diurnal mean OPP is a risk factor for the diagnosis of POAG [9]. Choi et al. reported that marked circadian mean OPP fluctuations might be a risk factor for the development of NTG [6]. Later, Choi et al. reported that a greater circadian mean OPP is significantly related to a decreased MD, an increased pattern SD, an increased Advanced Glaucoma Intervention Study (AGIS) score, a reduced temporal, superior, nasal, inferior, and temporal (TSNIT) average, a reduced inferior average, and an increased nerve fibre indicator on scanning laser polarimetry [7]. Sung et al. reported on 101 NTG patients who underwent 24-h sitting IOP and OPP measurements over more than 4 years. The 24-h OPP fluctuations were found to be significant predictors of VF progression [8]. Therefore, OPP has been found to be a risk factor for the severity and progression of glaucoma in previous studies. In the present study, the IOP/OPP fluctuations in the glaucoma patients were not highly reproducible. Only the peak OPP of the POAG patients occurred at same time point (24:00) in the diurnal curves from the 2 consecutive days. Previous studies might not have comprehensively demonstrated the relevance of IOP/OPP to glaucoma. Moreover, different equations have been used to calculate the OPP; OPP has also been defined as ([DBP-1/3 (SBP-DBP)]-IOP) [21]. Different methods of calculation would lead to different results. Twenty-four-hour OPP fluctuations may be associated with nocturnal BP reductions [8, 28]. We did not analyse the relationship between the reproducibility of OPP fluctuations and nocturnal BP reductions. Further studies are needed to identify the factors that influence the reproducibility of OPP measurements.

There are some limitations in this study. We did not measure the IOP at 3:00 over the 2 consecutive days. This measurement would have required us to wake up the subjects, which would have disturbed their sleep-wake rhythm. This strategy might have affected the IOP and BP measurements at the later time points. Recently, a wireless contact lens sensor (CLS) was used to measure IOPs over 24 h, and this approach did not require the waking of sleeping subjects [29]. Applanation resonance tonometers are less affected by corneal properties than GATs and exhibit good inter-examiner reproducibility and intra-examiner repeatability [30]. Short-term IOP measurements and long-term IOP measurements will be more comfortably and easily realized in the future. All of the IOP and BP measurements were not performed by a single doctor, but the IOP and BP measurements within each day were performed by single experienced doctors.

## Conclusion

The IOP measurements within a single day were not highly reproducible in the short-term. This report is the first to document the reproducibility of OPP measurements in a different study population. The OPP measurements exhibited better reproducibilities than the IOP measurements. The normal volunteers exhibited better IOP and OPP reproducibilities than the glaucoma patients. The IOP and OPP fluctuations exhibited fair or poor reproducibilities; therefore, IOP and OPP fluctuations cannot be assessed based on IOP and OPP measurements collected within a single day.

## Abbreviations

AGIS: Advanced glaucoma intervention study
BP: Blood pressure
CCT: Central corneal thickness
CLS: Contact lens sensor
DBP: Diastolic blood pressure
GAT: Goldmann applanation tonometer
HR: Heart rate
ICCs: Intraclass correlation coefficients
IOP: Intraocular pressure
MD: Mean deviation
NTG: Normal-tension glaucoma
OHT: Ocular hypertension
OPP: Ocular perfusion pressure
POAG: Primary open-angle glaucoma
SBP: Systolic blood pressure
TSNIT: Temporal, superior, nasal, inferior, and temporal
VF: Visual field
